# Draft Genome Sequence of Marine-Derived Streptomyces sp. TP-A0873, a Producer of a Pyrrolizidine Alkaloid Bohemamine

**DOI:** 10.1128/genomeA.00008-15

**Published:** 2015-02-19

**Authors:** Hisayuki Komaki, Natsuko Ichikawa, Akira Hosoyama, Nobuyuki Fujita, Yasuhiro Igarashi

**Affiliations:** a Biological Resource Center, National Institute of Technology and Evaluation (NBRC), Chiba, Japan; b NBRC, Tokyo, Japan; c Biotechnology Research Center and Department of Biotechnology, Toyama Prefectural University, Toyama, Japan

## Abstract

Streptomyces sp. TP-A0873, isolated from deep-sea water, produces three different classes of secondary metabolites: antimycin, bohemamine, and alkylated butenolides. In order to assess the biosynthetic potential of this strain, draft genome sequencing was carried out. The genome contained at least 14 gene clusters for polyketide synthase (PKS) and nonribosomal peptide synthetase (NRPS).

## GENOME ANNOUNCEMENT

Marine actinomycetes are promising sources of novel bioactive compounds with high chemical and biological diversity (1–3). In our screening program for bioactive secondary metabolites from marine-derived actinomycetes, Streptomyces sp. TP-A0873 collected from deep-sea water in Toyama Bay, Japan, was found to produce antimycins, bohemamins, and alkylated butenolides (4). To assess the capacity of this strain in secondary metabolism, we carried out whole-genome shotgun sequencing of strain TP-A0873 and analyzed the polyketide synthase (PKS) and nonribosomal peptide synthetase (NRPS) gene clusters in the genome.

Streptomyces sp. TP-A0873 was deposited at the NBRC culture collection with the registration number of NBRC 110035. The whole genome of strain TP-A0873 monoisolate was read by using a combined strategy of shotgun sequencing with GS FLX+ (Roche) (48-Mb sequences, 6-fold coverage) and paired-end sequencing with HiSeq1000 (Illumina) (678 Mb, 90-fold coverage). These reads were assembled using Newbler v2.6 software, and subsequently finished using GenoFinisher software (5), which led to a final assembly of 89 scaffold sequences of >500 bp each. The total size of the assembly was 7,508,934 bp, with a G+C content of 71.7%. Coding sequences were predicted by Prodigal (6). PKS and NRPS gene clusters were identified in the same manner as previously described (7). The genome contained at least three type I PKS, three type II PKS, four NRPS, and four hybrid PKS/NRPS gene clusters. One of the type I PKS gene clusters was partial and fragmented into nine scaffolds/contigs but was characterized as a homolog of an *fsc* cluster responsible for the production of polyene macrolide (8). The type II PKS gene cluster in scaffold02 was predicted as a biosynthetic gene cluster for cosmomycin-like aromatic compounds. Scaffold23 encoded two type II PKS gene clusters, one of which is likely responsible for spore pigment synthesis. The hybrid PKS/NRPS gene cluster in scaffold05 including orf119 (*antC*) and orf118 (*antD*) was characterized as a gene cluster for antimycin biosynthesis (9). Another hybrid PKS/NRPS gene cluster in scaffold01 was similar to the *lnm* cluster (10, 11) but the domain organization was not completely identical, suggesting the possibility of production of new leinamycin derivatives. At present, biosynthetic gene clusters for bohemamine and butenolides have not been identified.

The genome sequence data of strain TP-A0873 suggested its high potential in secondary metabolism and gave important information for the discovery of new bioactive compounds. Further detailed inspection of the genomic data may disclose the biosynthetic genes for bohemamine, a unique alkaloid for which the biosynthesis mechanisms remain unknown.

### Nucleotide sequence accession numbers.

This whole-genome sequence has been deposited in DDBJ/ENA/GenBank under the accession no. BBNN00000000. The version described in this paper is the first version, BBNN01000000.

## References

[1] Fenical W, Jensen PR 2006 Developing a new resource for drug discovery: marine actinomycete bacteria. Nat Chem Biol 2:666–673. doi:10.1038/nchembio841. 17108984

[2] Jensen PR, Mincer TJ, Williams PG, Fenical W 2005 Marine actinomycete diversity and natural product discovery. Antonie Van Leeuwenhoek 87:43–48. doi:10.1007/s10482-004-6540-1. 15726290

[3] Lam KS 2006 Discovery of novel metabolites from marine actinomycetes. Curr Opin Microbiol 9:245–251. doi:10.1016/j.mib.2006.03.004. 16675289

[4] Igarashi Y, Ikeda M, Miyanaga S, Kasai H, Shizuri Y, Matsuura N 2014 11 12 Two butenolides with PPARα agonistic activity from a marine-derived *Streptomyces*. J Antibiot doi:10.1038/ja.2014.151. 25388602

[5] Ohtsubo Y, Maruyama F, Mitsui H, Nagata Y, Tsuda M 2012 Complete genome sequence of *Acidovorax* sp. strain KKS102, a polychlorinated-biphenyl degrader. J Bacteriol 194:6970–6971. doi:10.1128/JB.01848-12. PMC3510582 23209225

[6] Hyatt D, Chen GL, Locascio PF, Land ML, Larimer FW, Hauser LJ 2010 Prodigal: prokaryotic gene recognition and translation initiation site identification. BMC Bioinformatics 11:119. doi:10.1186/1471-2105-11-119. 20211023 PMC2848648

[7] Komaki H, Ichikawa N, Hosoyama A, Fujita N, Igarashi Y 2014 Draft genome sequence of marine-derived actinomycete *Nocardiopsis* sp. strain TP-A0876, a producer of polyketide pyrones. Genome Announc 2 (4):e00665-14. doi:10.1128/genomeA.00665-14. PMC4110758 25013138

[8] Chen S, Huang X, Zhou X, Bai L, He J, Jeong KJ, Lee SY, Deng Z 2003 Organizational and mutational analysis of a complete FR-008/candicidin gene cluster encoding a structurally related polyene complex. Chem Biol 10:1065–1076. doi:10.1016/j.chembiol.2003.10.007. 14652074

[9] Yan Y, Zhang L, Ito T, Qu X, Asakawa Y, Awakawa T, Abe I, Liu W 2012 Biosynthetic pathway for high structural diversity of a common dilactone core in antimycin production. Org Lett 14:4142–4145. doi:10.1021/ol301785x. 22861048

[10] Cheng YQ, Tang GL, Shen B 2002 Identification and localization of the gene cluster encoding biosynthesis of the antitumor macrolactam leinamycin in *Streptomyces atroolivaceus* S-140. J Bacteriol 184:7013–7024. doi:10.1128/JB.184.24.7013-7024.2002. 12446651 PMC135466

[11] Cheng YQ, Tang GL, Shen B 2003 Type I polyketide synthase requiring a discrete acyltransferase for polyketide biosynthesis. Proc Natl Acad Sci U S A 100:3149–3154. doi:10.1073/pnas.0537286100. 12598647 PMC152261

